# Patient Navigation Programs in Canada for People With Dementia, Their Caregivers and Care Providers: An Environmental Scan

**DOI:** 10.1177/30495334261446734

**Published:** 2026-05-05

**Authors:** Grailing Anthonisen, Alison Luke, Pat Charlton, Shelley Doucet

**Affiliations:** 1 University of New Brunswick – Saint John, NB, Canada; 2 University of Prince Edward Island, Charlottetown, PE, Canada

**Keywords:** dementia, community, health services research, qualitative methodology, care partner

## Abstract

As the rates of dementia continue to rise across Canada, accessing care remains a challenge for people living with dementia, their caregivers, and members of the care team. Patient navigation offers a promising approach to reducing these barriers and enhancing the integration of care for this population. Many such programs already exist in Canada. This scan identified 11 programs across six provinces, detailing their characteristics, implementation processes, facilitators, and barriers to their implementation. Findings highlight the importance of person-centered care, early access, effective collaboration, and adequate funding as key facilitators. In contrast, barriers included insufficient funding, insufficient capacity for collaboration, and system-level challenges, like “navigation to nowhere” and disruptions caused by COVID-19. By reporting on successful practices and areas for improvement, this scan aims to inform the development and implementation of effective dementia patient navigation programs.

## Introduction

Dementia is an umbrella term that describes a range of symptoms associated with cognitive impairment and decline (Alzheimer’s Association, 2023; Wong et al., 2016). It can cause difficulty thinking, reasoning, remembering, and making decisions, as well as changes in behavior and abilities that impede daily life (Alzheimer’s Association, 2023; Wong et al., 2016). The term dementia covers several conditions, such as Alzheimer’s disease, vascular dementia, and Lewy body dementia, among others. While the risk of dementia increases with age, dementia is not part of healthy aging (Alzheimer’s Association, 2023). People living with dementia have complex care needs that change as their symptoms progress, requiring significant care and support from their caregivers (i.e., family, friends, or neighbors; Wong et al., 2016). They are also more likely to require the care of multiple providers and use health care services more frequently (Ashbourne et al., 2021).

As rates of dementia increase across Canada, people living with dementia, their caregivers, and the care team can face many barriers. These can include stigma and denial (Canadian Academy of Health Sciences, 2018); a lack of knowledge and information about dementia and the available services (Canadian Academy of Health Sciences, 2018); limited access to health and social care (Hill et al., 2021); and services that don’t meet their needs (Canadian Academy of Health Sciences, 2018; Peel & Harding, 2014). Caregivers frequently experience burnout; high rates of stress; poor mental health; lower levels of subjective well-being; and poor medical outcomes (Alzheimer’s Association, 2023). These issues are exacerbated by fragmented health and social care systems that are difficult to navigate (Canadian Academy of Health Sciences, 2018; Peel & Harding, 2014). Regardless of whether they have a diagnosis, people with dementia often do not receive the most appropriate services (OECD, 2018). Increasing access to resources, information, and services, as well as supporting and building the capacity of caregivers and care team members, are vital to enhancing the quality of care (Public Health Agency of Canada, 2019; Public Health Agency of Canada, 2023).

Patient navigation is one way to address these challenges. By proactively connecting patients to resources, programs, and services that address their unmet needs, it eliminates barriers and provides support for individuals and their families as they navigate health and social care systems (Doucet et al., 2019). While patient navigation was initially implemented and developed to support African Americans living with breast cancer (Freeman & Rodriguez, 2011), it has since been adapted to support people with other conditions, such as mental illness (Diaz-Linhart et al., 2016), kidney disease (Scholes-Robertson et al., 2022), HIV (Roland et al., 2022), and recently dementia (Bernstein et al., 2019). Patient navigators provide this support in a variety of ways, including by providing education, coordinating care, and facilitating access to appropriate services and resources. Navigators can have professional backgrounds, such as in nursing or social work, lay backgrounds, or peer backgrounds, that is, someone who has lived experience either as a patient or caregiver (Doucet et al., 2019; Kelly et al., 2019). In Canada, patient navigation is becoming more common across health and social care systems in diverse care contexts (Kokorelias et al., 2023; Tang et al., 2021; Walkinshaw, 2011). Canada’s navigation landscape mirrors international trends of attempting to reduce care fragmentation, while its programs are shaped by geographic and jurisdictional variations, with services advancing goals of personalized and coordinated care through models that are adapted to local circumstances (Champ & Dixon, 2024; Rabi et al., 2025).

Because patient navigation focuses on reducing barriers and promoting integrated care, it has the potential to benefit people living with dementia, their caregivers, and the care team (Anthonisen et al., 2023; Kokorelias et al., 2023; Spiro et al., 2012). Many such programs already exist in Canada. However, despite this growth, there remains limited documentation on the characteristics, implementation processes, barriers, and facilitators of dementia navigation programs in the Canadian context (Anthonisen et al., 2023; Kokorelias et al., 2023). Existing literature is well elaborated for cancer care, with less focus on other care contexts (Carter et al., 2018; McBrien et al., 2018; Valaitis et al., 2017). As for dementia and patient navigation, there are more examples from the American context, leaving a gap in understanding what programs exist in Canada and how they are adapted to meet the unique needs of dementia care. This scan addresses that gap by identifying the characteristics, implementation processes, barriers, and facilitators of current dementia patient navigation programs in Canada. These findings can provide guidance to healthcare professionals, organizations, and decision-makers on the development of patient navigation programs, or similar supports, for this population.

## Methods

### Design

Environmental scans gather information from external environments to inform decision-making and future action on a particular topic (Choo, 2001). They are commonly implemented to examine the current landscape of programs, policies, and services, and are an effective tool to inform health policy and practice, including program design (Charlton et al., 2021). Drawing on Charlton et al.’s (2021) scoping review of environmental scans of health services delivery, we adopted a mixed active and passive data collection approach, while also drawing on existing resources, such as CADTH scan, as a starting point. This reflects the combination of person and non-person data sources, which are common in scans of this type. Our environmental scan employed a mixed methods approach and incorporated a survey, analyzed primarily using descriptive statistics and, and semi-structured interviews, analyzed using qualitative content analysis. We conducted this environmental scan from February to August 2021 to identify existing navigation programs in Canada, describe their characteristics, implementation processes, and identify barriers and facilitators to their implementation. This scan was updated between July and August 2024.

### Research Questions

We addressed the following questions:

- **Q1**: What are the characteristics, logistical and otherwise, of patient navigation programs across Canada for people with dementia, their caregivers, and members of the care team?
- **Q2**: What are the barriers and facilitators to the operation and implementation of such programs?

### Program Identification

We identified programs using multiple strategies. As a starting point, we drew on a scan by the Canadian Agency for Drugs and Technologies in Health, which included both dementia-specific and more general navigation programs (Banerjee & Argáez, 2021). We also identified programs from a general Google search using the search terms *patient navigation, dementia, cognitive decline, cognitive dysfunction*, and *Alzheimer’s*. We sent email inquiries to these organizations to ascertain if they offered navigation services for people with dementia, their caregivers, or the care team, or if they knew of any such programs. We also contacted 20 additional organizations that served older adults or navigation programs targeting other populations to ask if they knew of existing patient navigation programs for people with dementia in their regions. In total, we contacted 49 organizations, and 34 organizations confirmed that they provided relevant services. Using Qualtrics, we distributed a survey to obtain data about their programs and then asked if key informants from programs would be willing to participate in an interview about implementation experiences. When the scan was updated, we contacted all participating programs again by email to confirm or update data. Ethics approval was provided by the University of New Brunswick Ethics Board in 2021 (REB File #005-2021).

### Survey Inclusion Criteria and Design

We included organizations in the environmental scan if they: (1) provided patient navigation for people with dementia, their caregivers, and/or the care team; (2) had a specific position designated to perform the navigation role (whether paid or volunteer); and (3) were based in Canada. Though 34 programs initially self-identified as patient navigation programs for this population, 23 did not meet the established criteria.

The survey consisted of 26 questions about the characteristics, implementation process, structure, and target population of the navigation programs. It contained both forced choice and open-ended questions. While the survey’s open-ended questions used free-text response formats, the questions mainly sought answers that were descriptive and factual (like, languages of service and position titles). For those other open-ended questions, we analyzed qualitatively. We developed a data extraction form using Excel based on the survey questions.

### Interview Criteria and Design

We conducted seven semi-structured interviews over Zoom with stakeholders who indicated at the end of the survey that they were willing to be contacted for further information, meaning the survey inclusion criteria also applied to the interviews. The interviews were 20 to 45 min and included open-ended questions and probes for possible follow-up questions. The questions focused on participants’ experiences delivering patient navigation programs for people with dementia, as well as barriers and facilitators to the development and implementation of their navigation programs. We also asked participants if they knew of any other patient navigation programs that served people with dementia, their caregivers, and members of the care team.

### Analysis

We coded and analyzed the survey data using the SAS statistical program. The quantitative analysis included descriptive statistics of the survey responses. Open ended questions were analyzed using qualitative content analysis (Mayring, 2000; Vaismoradi et al., 2013).

All interviews were transcribed verbatim and reviewed for accuracy. We conducted a qualitative content analysis using Excel to organize the coding process (Vaismoradi et al., 2013). Two of the authors reviewed and coded the interview transcripts independently, using an inductive approach, recording ideas and patterns in the data. Coding discrepancies were addressed through discussion and consensus. Themes were collaboratively collated and developed from these codes, then refined and clearly defined through further discussion before being finalized.

## Results

### Survey Results

We identified 11 patient navigation programs for people with dementia, their caregivers, and/or the care team. Programs were in Alberta (*n* = 3), British Columbia (*n* = 3), New Brunswick (*n* = 1), Ontario (*n* = 3), Prince Edward Island (*n* = 1), and Saskatchewan (*n* = 1), with one program spanning two provinces. Eight organizations were non-profit, two were government-run programs, and one was a for profit program. Six of the programs were associated with provincial Alzheimer Societies’ First Link programs. Eight of the navigation programs were launched since 2012. Table 1 outlines the organizations providing services, their organization models, and populations served.

**Table 1.** Organization Demographics & Population Served.

#	Navigation service name	Service location	Organization that provides services	Type of organization	Navigation service start date	Target population
1	Dementia care coach	Ottawa & Renfrew County in Ontario	The dementia society of Ottawa and Renfrew county	Non-profit organization	1980	Caregivers
2	Dementia care team	Calgary, Alberta	Alberta health services	Government	October 2012	People w/dementia (mostly age 65+)
3	Dementia care team integrated home care	Calgary, Alberta	Alberta health services	Government	2012	People w/dementia; caregivers (target age 65 + but accept younger)
4	First link care navigation w/Alzheimer societies	Across Ontario – 26 chapter locations	Alzheimer society of Ontario – 26 chapters	Non-profit organization	2017	People w/dementia; caregivers
5	First link dementia helpline	British Columbia	Alzheimer society of British Columbia	Non-profit organization	Unsure (at least 10 years ago)	People w/dementia; caregivers; care providers
6	First link dementia support	British Columbia	Alzheimer society of British Columbia	Non-profit organization	January 2010	People w/dementia; caregivers
7	First link PEI	Prince Edward Island	Alzheimer society of Prince Edward Island	Non-profit organization	2013	People w/dementia; caregivers
8	First link program	Saskatchewan	Alzheimer society of Saskatchewan	Non-profit organization	October 2019	People w/dementia; caregivers
9	First link w/Alzheimer society of New Brunswick – Fredericton chapter	Fredericton/Moncton/Saint John/Tracadie/Edmundston, NB	Alzheimer society of New Brunswick	Non-profit organization	Unsure	People w/dementia; caregivers; care providers
10	Proactive seniors dementia care coaching	Alberta & Kelowna, British Columbia	Proactive seniors	Private consulting company	2016	Caregivers
11	Regional MINT memory clinic	Kitchener, Ontario	Center for family medicine	Non-profit organization	April 2012	Caregivers (age 65+)

#### Populations Served

The programs identified in this scan varied in terms of their target populations. One program was designed specifically for people with dementia, while three were focused on supporting caregivers. Five programs targeted people with dementia and their caregivers. Two programs served care providers in addition to people with dementia and their caregivers. Most of these programs (*n* = 9) did not target a specific age group.

#### Logistical Characteristics of Programs

The programs provided access to their services in multiple ways, and as a result, several programs are included in more than one service delivery category (see Table 2 for full details). The most common methods of service delivery included telephone (*n* = 10); web-based platforms (*n* = 8); in-person services provided in office or clinic (*n* = 7); and home visits or other outreach (*n* = 6; see Table 2). One program did not offer any services in-person.

**Table 2.** Logistical Characteristics of Programs & Evaluation of Programs.

#	Navigation delivery	Hours of operation	Languages available	Types of referrals	How families and caregivers learn about service
1	• Telephone • Web-based • In-person in office or clinic • Home visits or other outreach	Mon.–Thur. 8:30AM–8:00PM; Fri. and Sat. 8:30AM–4:00PM	English; Arabic; Spanish	Self-referral; physicians; other clinicians and health care providers	Word of mouth; their health care provider; advertisements
2	• Home visits or other outreach • Other: video conferencing	8:00AM–4:15PM	There is a translator service	Physicians; other clinicians and health care providers	Their health care provider; through home care
3	• Telephone • Home visits or other outreach	Mon.–Fri. 8:00AM to 4:15PM; weekends (8:00AM–4:15PM, by urgent request)	There is a translator service	Physicians; other clinicians and health care providers; other	Their health care provider
4	• Telephone • Web-based • In-person in office or clinic • Home visits or other outreach	Mon.–Fri. 9:00AM–5:00PM (with some variation at each chapter)	English; French at designated sites; additional languages varying by service location	Self-referral; physicians; other clinicians and health care providers; other	Word of mouth; their health care provider; advertisements; police referral; legal aid referral; other community support services
5	• Telephone • Web-based	English: Mon.–Fri. 9:00AM–8:00PM; Chinese and Punjabi: Mon.–Fri. 9:00AM to 4:00 PM	English; Cantonese; Mandarin; Punjabi	Self-referral; other	Word of mouth; their health care provider; advertisements
6	• Telephone • Web-based • In-person in office or clinic	Mon.–Fri. 9:00AM–5:00PM	English; Cantonese, Mandarin; Punjabi, Hindi, Urdu, Farsi	Physicians; other clinicians and health care providers	Word of mouth; their health care provider; advertisements
7	• Telephone • Web-based • In-person in office or clinic • Home visits or other outreach	Mon.–Fri. 8:30AM–4:30PM	English	Self-referral; physicians; other clinicians and health care providers	Word of mouth; their health care provider; advertisements
8	• Telephone • Web-based • In-person in office or clinic	8:30AM–12:15PM and 12:45PM–4:30PM	English	Physicians; other clinicians and health care providers; other: community organizations	Word of mouth; their health care provider; social media
9	• Telephone • Web-based • In-person in office or clinic	8:30AM–4:30PM	English; French	Self-referrals; physicians; other clinicians and health care providers	Word of mouth; their health care provider; advertisements
10	• Telephone • Home visits, other outreach • Other: video conference	Mon.–Fri. 8:00AM–6:00PM	English	Self-referral; physicians; other clinicians and health care providers	Word of mouth; their health care provider; advertisements
11	• Telephone • Web-based • In-person in office or clinic	8:30AM–4:30PM	English	Physicians; other clinicians and health care providers	Their health care provider

Most programs operated Monday to Friday during regular business hours (*n* = 7), while two programs also reported being open during weekday evenings. One program reported being open on Saturdays. Five programs offered services in languages other than English, some providing services in multiple languages. This included Punjabi (*n* = 3), French (*n* = 2), Cantonese (*n* = 2), Mandarin (*n* = 2), Arabic (*n* = 1) and Spanish (*n* = 1). Two programs also had translator services available, and one program did not specify which additional languages were offered.

The programs reported multiple sources of referrals to their services, with several counted in more than one referral category. Most programs (*n* = 10) received referrals from physicians and other clinicians and health care providers. Six programs received self-referrals. All programs reported that clients learned about their services from their healthcare provider. Eight programs reported word of mouth and seven reported using advertisements to inform clients, families, and caregivers about the services. A minority of programs reported other sources.

#### Description of Services Provided by Navigators

All programs reported providing emotional support to clients, as well as facilitating linkages to other community social supports. In all the programs, navigators identified barriers and assisted clients in addressing these barriers. Ten programs also reported building clients’ capacity by providing advice and support. Most (*n* = 10) programs provided tailored education and support to clients, as well as referrals to other care providers for assessments and consultations. Nine organizations assessed client needs for assistance and resources, while another nine also supported clients in providing a safe physical environment. The services navigators provided are described fully in Table 3.

**Table 3.** Description of Services.

#	Services provided by navigator	Types of info & education provided to caregivers
1	• Assess client/caregiver needs for assistance and resources • Identify barriers and assist client/caregiver in addressing these barriers • Provide tailored education and support to clients/caregivers • Provide emotional support to clients/caregivers • Build capacity of caregivers by providing advice and support (e.g., assistance with problem-solving stressful situations, effective communication and behavior management strategies, conflict management, dealing with crisis) • Provide advice and support on providing a safe physical environment • Provide referrals to other care providers for assessment or consultation • Coordinate access to services and resources • Provide assistance with making medical or other appointments • Provide assistance with transportation needs • Facilitate linkages to other community social supports • Provide assistance with dealing with financial concerns • Provide assistance with completing applications for funding or services • Advocate on behalf of the client or caregiver	• Info about the disease and disease progression • Info about additional community resources and supports • Info about advance care planning • Info and advice about effective communication strategies, and dealing with difficult behaviors • Info about care for the caregiver (e.g., maintaining healthy behaviors, self-care, and stress management)
2	• Assess client/caregiver needs for assistance and resources • Identify barriers and assist client/caregiver in addressing these barriers • Provide tailored education and support to clients/caregivers • Provide emotional support to clients/caregivers • Build capacity of caregivers by providing advice and support (e.g., assistance with problem-solving stressful situations, effective communication and behavior management strategies, conflict management, dealing with crisis) • Provide advice and support on providing a safe physical environment • Provide referrals to other care providers for assessment or consultation • Coordinate access to services and resources • Provide assistance with making medical or other appointments • Facilitate linkages to other community social supports • Advocate on behalf of the client or caregiver	• Info about the disease and disease progression • Info about additional community resources and supports • Info about advance care planning • Info and advice about effective communication strategies, and dealing with difficult behaviors • Info about care for the caregiver (e.g., maintaining healthy behaviors, self-care, and stress management)
3	• Assess client/caregiver needs for assistance and resources • Identify barriers and assist client/caregiver in addressing these barriers • Provide tailored education and support to clients/caregivers • Provide emotional support to clients/caregivers • Build capacity of caregivers by providing advice and support (e.g., assistance with problem-solving stressful situations, effective communication and behavior management strategies, conflict management, dealing with crisis) • Provide advice and support on providing a safe physical environment • Provide referrals to other care providers for assessment or consultation • Coordinate access to services and resources • Provide assistance with transportation needs • Facilitate linkages to other community social supports • Provide assistance with dealing with financial concerns • Provide assistance with identifying funding and other resources • Provide assistance with completing applications for funding or services • Advocate on behalf of the client or caregiver	• Info about the disease and disease progression • Info about additional community resources and supports • Info about advance care planning • Info and advice about effective communication strategies, and dealing with difficult behaviors • Info about care for the caregiver (e.g., maintaining healthy behaviors, self-care, and stress management)
4	• Assess client/caregiver needs for assistance and resources • Identify barriers and assist client/caregiver in addressing these barriers • Provide tailored education and support to clients/caregivers • Provide emotional support to clients/caregivers • Build capacity of caregivers by providing advice and support (e.g., assistance with problem-solving stressful situations, effective communication and behavior management strategies, conflict management, dealing with crisis) • Provide advice and support on providing a safe physical environment • Provide referrals to other care providers for assessment or consultation • Coordinate access to services and resources • Provide assistance with making medical or other appointments • Provide assistance with transportation needs • Facilitate linkages to other community social supports • Provide assistance with dealing with financial concerns • Provide assistance with identifying funding and other resources • Provide assistance with completing applications for funding or services • Advocate on behalf of the client or caregiver	• Info about the disease and disease progression • Info about additional community resources and supports • Info about advance care planning • Info and advice about effective communication strategies, and dealing with difficult behaviors • Info about care for the caregiver (e.g., maintaining healthy behaviors, self-care, and stress management)
5	• Identify barriers and assist client/caregiver in addressing these barriers • Provide tailored education and support to clients/caregivers • Provide emotional support to clients/caregivers • Build capacity of caregivers by providing advice and support (e.g., assistance with problem-solving stressful situations, effective communication and behavior management strategies, conflict management, dealing with crisis) • Provide referrals to other care providers for assessment or consultation • Facilitate linkages to other community social supports	• Info about the disease and disease progression • Info about additional community resources and supports • Info about advance care planning • Info and advice about effective communication strategies, and dealing with difficult behaviors • Info about care for the caregiver (e.g., maintaining healthy behaviors, self-care, and stress management)
6	• Assess client/caregiver needs for assistance and resources • Identify barriers and assist client/caregiver in addressing these barriers • Provide tailored education and support to clients/caregivers • Provide emotional support to clients/caregivers • Build capacity of caregivers by providing advice and support (e.g., assistance with problem-solving stressful situations, effective communication and behavior management strategies, conflict management, dealing with crisis) • Provide advice and support on providing a safe physical environment • Provide referrals to other care providers for assessment or consultation • Facilitate linkages to other community social supports • Provide assistance with dealing with financial concerns	• Info about the disease and disease progression • Info about additional community resources and supports • Info about advance care planning • Info and advice about effective communication strategies, and dealing with difficult behaviors • Info about care for the caregiver (e.g., maintaining healthy behaviors, self-care, and stress management)
7	• Assess client/caregiver needs for assistance and resources • Identify barriers and assist client/caregiver in addressing these barriers • Provide tailored education and support to clients/caregivers • Provide emotional support to clients/caregivers • Build capacity of caregivers by providing advice and support (e.g., assistance with problem-solving stressful situations, effective communication and behavior management strategies, conflict management, dealing with crisis) • Provide advice and support on providing a safe physical environment • Provide referrals to other care providers for assessment or consultation • Facilitate linkages to other community social supports	• Info about the disease and disease progression • Info about additional community resources and supports • Info about advance care planning • Info and advice about effective communication strategies, and dealing with difficult behaviors • Info about care for the caregiver (e.g., maintaining healthy behaviors, self-care, and stress management)
8	• Assess client/caregiver needs for assistance and resources • Provide emotional support to clients/caregivers • Build capacity of caregivers by providing advice and support (e.g., assistance with problem-solving stressful situations, effective communication and behavior management strategies, conflict management, dealing with crisis) • Provide advice and support on providing a safe physical environment • Provide referrals to other care providers for assessment or consultation • Facilitate linkages to other community social supports • Provide assistance with identifying funding and other resources	• Info about the disease and disease progression • Info about additional community resources and supports • Info about advance care planning • Info and advice about effective communication strategies, and dealing with difficult behaviors • Info about care for the caregiver (e.g., maintaining healthy behaviors, self-care, and stress management)
9	• Identify barriers and assist client/caregiver in addressing these barriers • Provide tailored education and support to clients/caregivers • Provide emotional support to clients/caregivers • Build capacity of caregivers by providing advice and support (e.g., assistance with problem-solving stressful situations, effective communication and behavior management strategies, conflict management, dealing with crisis) • Facilitate linkages to other community social supports	• Info about the disease and disease progression • Info about additional community resources and supports • Info about advance care planning • Info and advice about effective communication strategies, and dealing with difficult behaviors • Info about care for the caregiver (e.g., maintaining healthy behaviors, self-care, and stress management)
10	• Assess client/caregiver needs for assistance and resources • Identify barriers and assist client/caregiver in addressing these barriers • Provide tailored education and support to clients/caregivers • Provide emotional support to clients/caregivers • Build capacity of caregivers by providing advice and support (e.g., assistance with problem-solving stressful situations, effective communication and behavior management strategies, conflict management, dealing with crisis) • Provide advice and support on providing a safe physical environment • Provide referrals to other care providers for assessment or consultation • Coordinate access to services and resources • Facilitate linkages to other community social supports • Provide assistance with identifying funding and other resources • Provide assistance with completing applications for funding or services • Advocate on behalf of the client or caregiver	• Info about the disease and disease progression • Info about additional community resources and supports • Info about advance care planning • Info and advice about effective communication strategies, and dealing with difficult behaviors • Info about care for the caregiver (e.g., maintaining healthy behaviors, self-care, and stress management)
11	• Assess client/caregiver needs for assistance and resources • Identify barriers and assist client/caregiver in addressing these barriers • Provide tailored education and support to clients/caregivers • Provide emotional support to clients/caregivers • Build capacity of caregivers by providing advice and support (e.g., assistance with problem-solving stressful situations, effective communication and behavior management strategies, conflict management, dealing with crisis) • Provide advice and support on providing a safe physical environment • Provide referrals to other care providers for assessment or consultation • Coordinate access to services and resources • Facilitate linkages to other community social supports • Advocate on behalf of the client or caregiver	• Info about the disease and disease progression • Info about additional community resources and supports • Info about advance care planning • Info and advice about effective communication strategies, and dealing with difficult behaviors • Info about care for the caregiver (e.g., maintaining healthy behaviors, self-care, and stress management)

All 11 programs provided education and informational support on advanced care planning and additional community resources. This included education on dementia and disease progression, support for caregivers (e.g., maintaining healthy behaviors, self-care, and stress management), and advice about effective communication strategies and dealing with difficult behaviors.

#### Navigator Title, Qualifications, Role, and Caseload

Navigator position titles varied, including across Alzheimer Society First Link programs. These titles included coordinator (*n* = 4), case manager (*n* = 2), navigator (*n* = 1), social worker (*n* = 1), among others (see Table 4). All were paid positions.

**Table 4.** Navigator Qualifications and Caseload.

#	Navigator position title	Is the navigator part of an integrated team?	Navigator qualifications	Navigator training	Full-time versus part-time (# of navigators)	Are the navigator positions paid?	Monthly max. caseload
1	Dementia care coach	No (has supervisor or other counterpoint)	No specific professional qualifications	Yes internal & external shadowing; GPA (Gentle Persuasive Approaches); DementiAbility; Positive Approach to Care; ASIST (Applied Suicide Intervention Skills Training)	Full-time (>3)	Yes	40 Active and 60 Passive
2	Dementia care team case managers	Yes (social workers, nurses, occupational therapists)	Nurse	Yes Dementia specific education	Full-time (>3)	Yes	30–40
3	Registered nurse case manager	Yes (social workers, nurses, occupational therapists)	Nurse; degree in a health-related field	Yes	Full-time (>3)	Yes	Varies
4	First link care navigator	Yes (other members vary by site)	Social worker; nurse; registered professional all strongly encouraged	Yes PIECES Training; Orientation to role; other as available including Anti-Racism; Lean Methodology; Advance Care Planning; Privacy; Consent & Capacity; etc.	Full-time & part-time (>3)	Yes	Target client served per year is 200. Monthly caseload varies.
5	Dementia helpline coordinator	No (has supervisor or other counterpoint)	No specific professional qualifications	Yes 4-month onboarding process learning about dementia; attending education sessions and other program sessions; shadowing existing staff; then being shadowed.	Full-time (>3)	Yes	No caseload
6	Support and education coordinator	No (has supervisor or other counterpoint)	No specific professional qualifications	Yes comprehensive support and education coordinator training; ongoing continuous education.	Full-time & part-time (23)	Yes	Don’t use a caseload system
7	Support services coordinator	Yes (social workers, nurses, psychologists, primary care providers, geriatricians)	Nurse; social worker; other degree in a health-related field	Yes	Full-time (>3)	Yes	48–60
8	Client services team manager	No	Social worker	Yes MHFA-OA (Mental Health First Aid for Older Adults); ASIST (Applied Suicide Intervention Skills Training). Art of Facilitation (Dialog Education Training) ASOS provides opportunity for Leadership Training, required DEI training on bias and anti-racism.	Full-time (2)	Yes	New clients per navigator approx. 25 plus carryover of at min an additional 10 (total min 35)
9	First link coordinator, first link support	No (has supervisor or other counterpoint)	No specific professional qualifications	Yes Manuals on First Link procedures	Full-time (1)	Yes	Unknown, sometimes over 250 follow-ups a week
10	Seniors advisor	Yes (seniors advisor team)	Degree in a health-related field (BA, MSc Aging and Health [Advisor 1], BSc Physical Therapy [Advisor 2])	Yes option to take various training programs	Part-time (2)	Yes	N/A
11	Social worker	Yes (social workers, nurses, primary care providers)	Social worker	Yes	Full-time (1) & part-time (1)	Yes	18 patients/month

Six programs reported that the navigator(s) worked as part of an integrated team to deliver services. The most common types of care providers on the integrated team were social workers (*n* = 4) and nurses (*n* = 4). Other team members include primary care providers (*n* = 2) and occupational therapists (*n* = 2).

While the qualifications for navigator positions varied, most required an advanced degree. The most common was a degree in social work (*n* = 4), nursing (*n* = 4), or degree in another health-related field (*n* = 3), with four organizations listing multiple backgrounds. The qualifications also varied among the Alzheimer Society First Link programs. All programs reported that they provided training for their navigators. Training varied widely (see Table 4). Training included topics such as dementia specific education, case management, Applied Suicide Intervention Skills Training (ASIST), and anti-racism training.

Seven organizations reported having more than three navigators, three programs reported having two navigators, and two reported having one navigator. Of the 11 programs, 7 had full-time navigators only, while 1 program had part-time navigators only.

There was not a standard method of measuring caseload across the included programs. Navigators measured caseloads in several ways: one reported number of follow-ups (over 250 per week), another measured number of patients served per month (18), and five reported caseload numbers (with one program reporting 30–32 cases and another reporting 30–40, e.g.). Table 4 describes navigator title, qualifications, role, and caseload in full.

### Interview Results

Seven key informants from six different organizations agreed to participate in interviews to share their perspectives on the barriers and facilitators affecting programs. Participants were based in Ontario (*n* = 2), New Brunswick (*n* = 2), PEI (*n* = 1), and Alberta (*n* = 1), with one participant representing a program available in both Ontario and Alberta. Each participant represented a distinct program apart from the New Brunswick participants, who were affiliated with the same initiative. Inductive qualitative content analysis produced three themes describing program facilitators, as well as four describing barriers (see Table 5).

**Table 5.** Themes and Sub-Themes.

Themes	Person-centered approach	The “well-oiled machine”	Successful care coordination and collaboration across sectors	“You start to feel the pressure”	“You know, it comes down to time and money, right?”	Lack of capacity for care coordination	Navigation to nowhere
Sub-themes	• Adapting supports to clients’ dementia journey • Early access	• Organizational setup • Ongoing training and skill development • Work-life balance		• Large and frequently increasing caseloads • System changes due to the COVID-19 pandemic • “A total moving target”	• Expanding services and professional development • Organizational strain caused by insufficient funding • Outreach		

#### Facilitators

The facilitator themes were: (1) Person-centered approach; (2) The “well-oiled machine”; and (3) Successful care coordination and collaboration.

##### Person-Centered Approach

Because the needs of people with dementia and their caregivers change over time, they require a variety of services, programs, and resources throughout their care journeys. This journey will vary according to their needs, as well as their individual wishes. This theme describes what care and support look like within this highly individualized context. It includes adapting support to clients’ dementia journey and early access to support.

##### Adapting Supports to Clients’ Dementia Journey

Multiple participants referred to seeking and receiving care as a “journey.” As one participant explained: “moving with them [people with dementia and their caregivers] along the journey is tremendously helpful” (03), while another noted that “they need someone to hold their hand and guide them through it (06).” While it was dementia that set them on this journey, not everyone will have the same beginning or ending with regard to their needs for services, resources, and/or programs. As such, many participants noted the importance of person-centered approaches in providing care. One participant described person-centered care planning as, “meeting people where they’re at (04).” While people with dementia and their caregivers needed support throughout this journey, clients did not need continuous support. That is, they were unlikely to need support at all times, but rather at specific points.

##### Early Access

Early access facilitated navigators in addressing the needs of people with dementia by enabling their involvement in decision-making about their care. This allowed support to be more inclusive of people with dementia. One participant explained: “early intervention provides the opportunity for individuals living with dementia to have a voice and planning for their own care while they’re still able to do so (01).” Early intervention provided the opportunity for people with dementia to have more agency and help set the course of their dementia journey.

##### The “Well-Oiled Machine.”

This theme describes the logistical aspects of PN, describing how organizational setup, ongoing training and skill development, and work-life balance acted as facilitators to program implementation.

##### Organizational Setup

Organizational setup refers to organizing navigator roles, responsibilities, and workload; allocating caseloads; specifying navigators’ tools and systems to assist clients; and establishing communication flows. As one participant put it, “So. . . you need a well-oiled machine with respect to, you know, being able to effectively receive referrals, (. . .) having really effective database, to capture client information (. . .; 03)..” Programs that support a cohesive team also facilitated this “well-oiled machine.” Training days, routine meetings, and open team communication encouraged co-learning and communal problem-solving. This helped address influxes of similar questions and challenges, such as due to the COVID-19 pandemic.

##### Ongoing Training and Skill Development

Ongoing training and skill development included the organization’s internal training, as well as accredited training courses. Internal training topics included dementia, community services, information on where to refer people, and how to handle a caseload. It was important that navigator knowledge be both specific to local contexts, while also spanning across sectors. As such, training and education was provided on an ongoing basis. One participant described how their organization provided annual training on cross-disciplinary best practices to update – or “boost” – all employees’ knowledge (07). Some organizations provided support interprovincially within their organization. Another organization provided training to ensure staff members’ knowledge and skillsets were consistent.

Additionally, there were a variety of skills that participants believed benefited patient navigation. Customer service was presented as a necessary skill and some navigators received explicit training on how to provide good service. Another skill identified was empathy. As one interviewee put it,

you need to have organized, empathetic staff (. . .) empathy, um, active listening, um, motivational interviewing, uh, and. . . knowledge of dementia and where people are at, and helping facilitate (. . .) the conversation so that you can come up with goals or co-create goals together (01).

For these programs, active listening skills were just as important as knowledge of dementia. Given that clients could be struggling, empathy was especially necessary. A human touch helped to deliver person-centered care during a journey that is so often challenging. When navigators possess these skills, it contributed to the smooth function and delivery of the program.

##### Work-Life Balance

Encouraging staff to have a work-life balance was crucial in preventing burnout. One participant commented on the importance of navigators taking care of their mental health and providing counseling and emotional support for the navigators. Another participant said:

We, uh, really try to, um, make sure that staff are, you know, balancing work-life and taking care of their mental health and um, we’ve dealt with a lot of changes during COVID just with our staff so just really trying to support them and meet them where they’re at too, because (. . .) sometimes it’s just ‘I had two really, really challenging calls’ and it’s like okay, you go outside, go for a walk for ten minutes, go get a coffee, do what you need to do you’ve got to take care of yourself because that’s what we tell our clients to do (04).

Work-life balance included setting clear boundaries around the navigator’s role. As one participant explained, it was outside of the navigators’ scope to handle mental health crises.

##### Successful Care Coordination and Collaboration Across Sectors

This theme highlights the importance of successful intersectoral and interdisciplinary collaboration as a key facilitator of effective care, allowing navigators and providers to circumvent system inefficiencies. Given the complexity of dementia, care often requires a coordinated, team-based approach. Participants described working within multidisciplinary teams and shared care models aimed to “take the hardest part out of dementia care” (07). While integrating navigation services into primary care improved system-wide collaboration and access, participants reported the difficulty of spreading and scaling patient navigation programs across primary care.

Collaboration and coordination also allowed navigators to draw on expertise outside the healthcare system, across sectors and professions. One participant worked with lawyers to assist clients with legal issues, such as power of attorney. Another participant commented on the importance of collaborating with community organizations like provincial or local Alzheimer Society chapters, as well as the government. These cross-sector partnerships are one strategy to work around systemic weakness, such as limited specialists and long wait times.

#### Barriers

The four themes describing barriers were: (1) “You start to feel the pressure”; (2) “You know, it comes down to time and money, right?”; (3) Lack of capacity for care coordination; and (4) Navigation to nowhere.

##### “You Start to Feel the Pressure.”

This theme describes the pressures navigators experienced, while providing support in a constantly changing system, including large, often-increasing caseloads, adapting to COVID-19-related system changes, and keeping track of available resources, services, and programs.

##### Large and Frequently Increasing Caseloads

Participants described the strain caused by growing caseloads, which they identified as a significant barrier to effective navigation. Many described their caseloads as a source of stress, which tended to grow with few opportunities to discharge patients. As one participant put it: “And so, you start to feel the pressure of (. . .) how many people can you navigate or manage on your caseload from a navigation perspective (03).” Navigators tended to add people to their caseload rather than discharge them. Each case represented a certain amount of work, even when the client was stable or doing well. This contributed to stress and concerns about sustainability over time.

##### System Changes Due to the COVID-19 Pandemic

The pandemic forced navigators to adapt how they delivered services. Prior to the pandemic, clients and navigators preferred meeting at the navigator’s office. One participant explained:

So, historically we did a lot of in-person work (. . .). And so, clients really did prefer to, to meet in-person at the office, (. . .) but COVID, and there was a reluctance, by both the clients and the staff, to leverage virtual engagement (. . .). Whether it’s Zoom, or what have you. So COVID forced our hand a little bit (03).

Because of the pandemic, there was also an influx of specific questions and a need for certain kinds of support, as more people experienced challenges related to their mental health and isolation. The pandemic also caused many support programs to be suspended or canceled. This reduction in available resources made it more difficult for navigators to connect clients with appropriate services, compounding the challenges already present in dementia care.

##### “A Total Moving Target:” Keeping on Top of Everything

Across interviews, participants discussed how difficult it was for navigators to keep track of all resources and programs in a constantly changing system. As one navigator explained,

You know it’s hard to keep up to date with all of this stuff, right? Like there will be a program that exists one day and then it doesn’t exist the next. There will be funding for something one day that’s not there the next. And so, you know it’s quite a challenge to, to keep on top and keep tabs of what’s happening and, (. . .), sometimes people are eligible for one thing and, other days they’re not. (. . .; 06)

Maintaining a clear picture of available supports and services meant that navigators needed a lot of specific knowledge of local systems and their communities. There was no universal or standard solution for all clients. One navigator explained, “even if you have this beautiful map laid out, (. . .) it’s not the same for every person, and every client” (04).

##### “You Know, It Comes Down to Time and Money, Right?”

This theme discusses how a lack of funding was a widespread barrier affecting navigation programs in a variety of ways, curtailing professional development, expansion of services, and outreach capacity.

##### Expanding Services and Professional Development

A lack of funding limited organizations’ capacity to provide and expand their services, which was important as dementia rates increased. Insufficient funding curtailed the duration and number of services that organizations and navigators offered. It also acted as a barrier to supporting clients, which is notable in the context of the progressive nature of dementia. An insufficient number of employees was a barrier to providing care throughout clients’ dementia journeys, as it limited the amount of follow-up navigators could provide clients. Being unable to hire more personnel limited existing navigators’ time and their availability to deliver services. Lack of funding also acted as a barrier to professional development for two different organizations as they could not afford to provide certain training programs for their navigators.

##### Organizational Strain Caused by Insufficient Funding

Whether programs were for-profit or non-profit, funding remained a challenge and created precarity. As one participant said, “the weakness obviously is, we can’t do it for free (. . .) we have to pay our staff and, and you have to have something to pay them with” (06). A participant at a non-profit program described “liv[ing] grant-to-grant” for the organization’s entire history (07). Another commented on the inconsistencies in funding from region to region in a province-wide model.

##### Outreach

While outreach and marketing could increase public awareness of navigation services, an absence of funding acted as a barrier. Families needed to be aware of the navigation services to utilize them. As one participant said, “the first challenge is actually getting people into your navigation system.” Participants reported that word-of-mouth was frequently the entry point for many families. This snowball effect was haphazard. As one participant explained, people may be aware of the organization “but they’re not familiar with what we do. And so that can actually prevent people from giving us a call” (01). Outreach may have its roots in systemic issues, such as limited infrastructure for public engagement or insufficient integration with primary care pathways. This impedes access to navigation services, suggesting that, while it may not be the sole cause, funding constraints may exacerbate outreach problem. Similarly, limitations on navigators’ time prevented them from investing more time and energy on outreach.

##### Lack of Capacity for Care Coordination

This theme describes a systemic barrier, the insufficient infrastructure supporting care coordination and collaboration impacting navigation programs. Navigators and care providers needed infrastructure to share information and records across sectors, including electronic medical records. Participants described this issue as a hindrance. Similarly, the system’s siloed structure also acted as a barrier. One navigator said,

So, when we look at, you know, as we hear across the world (. . .) not having a common, um, medical record for clients, uh, is problematic because th-the sharing of information isn’t seamless and you know there’s duplication. (. . .) It of course isn’t efficient and it certainly is not patient- or client-centred. Um, so, not having common EMR, is-is very problematic (03).

These problems are beyond navigators’ control, but were reported to impact their work, nonetheless.

##### Navigating to Nowhere

On a system level, the limited availability of support services was a significant barrier. Successful navigation depends on resources and services to direct clients to. The absence of such resources and services makes navigation impossible. Whereas the previous theme describes insufficient infrastructure and siloed communication between providers, this theme describes the absence of infrastructure, resources and services. As one participant described, “there’s just such limited services when we think about what’s available for individuals with dementia and their families” (01). Even when services existed, excessively long waitlists for these services could make referrals ineffective.

Geographic inconsistencies in available supports and services created additional challenges. Supports and services were not always available in rural settings, meaning there was nowhere for navigators to connect clients. As one interviewee described,

the inequity of supports and services across the province uh, and the variation does (. . .) create complexity to navigating because what’s available to support clients in one area isn’t in others, and, you know, sharing. . . best practices and-and resources is harder to do in that regard (03).

Participants commented on this unpredictability of funding and service availability, particularly when comparing urban and rural areas. This was especially challenging for province-wide organizations, where navigators had to adapt their approach based on geographic location, often without alternatives for clients in rural communities.

## Discussion

This environmental scan describes patient navigation programs across Canada that support people with dementia, their caregivers, and the care team. It highlights practices that can help to facilitate successful program implementation and identifies the barriers that may hinder it. We collected data on 11 programs, including eligibility requirements, hours and languages of operation, delivery methods, navigator title and background, services provided, as well as barriers and facilitators to program implementation.

Most of the programs identified in this scan did not impose age requirements. While some were designed for individuals 65 years and older, they still accepted younger clients. This flexibility in age requirements can improve access, particularly for those with early-onset dementia (Kuruppu & Matthews, 2013). The absence of strict age requirements differs from some of the existing literature on patient navigation programs for people with dementia, where age requirements are common, with clients often being required to enroll as dyads, (i.e., people with dementia with their caregivers; Anthonisen et al., 2023; Bass et al., 2013; Bernstein et al., 2019). Notably, several of these programs were also research studies, which must adhere to strict inclusion criteria when collecting data.

The programs identified by this scan provided a range of training opportunities for navigators. Although, navigation programs frequently provide ongoing training for growth and professional development, as well as motivational interview training (Battaglia et al., 2012; Cervantes et al., 2020; Valaitis et al., 2017), navigator training is not standardized across patient populations (Cantril & Haylock, 2013). Standardized training and skill development can help navigators set boundaries in the workplace and reduce burnout, which improves navigator retention (Roland et al., 2022). Navigation literature discusses the need to provide more training and support for navigators, particularly to develop skills in identifying existing services and best practices (Carter et al., 2017; Cervantes et al., 2020). Training, as well as navigator peer support, have the added benefit of improving retention (Carroll et al., 2018).

As such, there are opportunities for the development of national standards for patient navigation, especially around training. These standards could formalize core competencies for navigators and provide a minimum standard for qualifications and skills. A standard curriculum can include specialized dementia knowledge, cultural competency training, caregiver support and education, goal setting, interview and active listening skills, crisis intervention, as well as intersectoral and interdisciplinary knowledge to help address social and health needs. This could also include quality indicators for program assessment across regions and rural and urban areas.

This scan found that programs used a range of methods to measure caseload, which reflects literature findings (Anthonisen et al., 2023). While patient navigation caseloads have not been standardized, some recommendations exist. Considering the complexity of patients’ cases, patient resource needs, and frequency of navigator contact, one caseload recommendation was 20 to 50 cases (Meade et al., 2014). Navigator caseload has also been identified as an area for future research to establish how to best assess and manage caseloads, to ensure patients receive optimal care (Freund, 2017; Meade et al., 2014). For other navigation programs, caseloads that were too large, overwhelmed navigators, decreasing quality of care (Raut et al., 2015). Standardized caseloads create an element of predictability that can promote efficiency, while ensuring that patients receive the right level of care (Freund, 2017).

The importance of providing person-centered care came up repeatedly across interviews. Person-centered care focuses on an individual’s unique needs, preferences, and background, allowing them to actively participate in their care (Santana et al., 2018). The variety of ways navigators in this scan communicated with clients reflects this flexibility, also demonstrated in the literature (Anthonisen et al., 2023; Fæø et al., 2020; Liu et al., 2021). Additionally, research reflects this scan’s finding that empathy is integral to navigators’ ability to deliver effective care (Bernstein et al., 2019). Strong listening skills and ensuring that people feel heard are crucial to providing meaningful support (Fæø et al., 2020). For navigators, interpersonal skills, such as empathy, are just as important as system knowledge (Roland et al., 2022). Empathetic, well-trained navigators can improve care outcomes (Cervantes et al., 2020).

This patient-centered care can help people with dementia maintain their sense of self and normalcy (Edvardsson et al., 2010). For example, person-centered care has resulted in a reduction of depressive symptoms (Molony et al., 2011) and an increase in quality of life for people with dementia (Kane et al., 2007; Koren, 2010). It can also benefit the organizations providing navigation services, as it allows them to address their own needs and goals (Riley & Riley, 2016). However, person-centered approaches need adequate personnel, training, and organizational support to be successful (Fossey et al., 2014; Lawrence & Kinn, 2012). This necessitates funding and personnel, which can be a challenge for programs, as this environmental scan found.

Interviews identified collaboration and coordination as program facilitators, which is consistent with literature on both general patient navigation and dementia-specific programs (Anderson & Larke, 2009a; Anthonisen et al., 2023; Valaitis et al., 2017). Dementia navigation programs highlighted collaboration with primary care providers, as well as between medical and community organizations (Anthonisen et al., 2023; Bass et al., 2013; Bernstein et al., 2019; Samus et al., 2015). Clearly defined roles based on patient needs can facilitate collaboration and prevent confusion or role duplication (Bryant-Lukosius & DiCenso, 2004). Collaborative dementia care has been associated with high satisfaction rates among care providers, people with dementia, and caregivers (Goldfarb et al., 2022), improved quality of life for people with dementia (Callahan et al., 2006; Counsell et al., 2006), better care outcomes (Heintz et al., 2020; Lee et al., 2014), and decreased caregiver burden (Callahan et al., 2006; Thyrian et al., 2017).

This scan also identified program implementation barriers that are consistent with those in the literature on other Canadian navigation programs (Carter et al., 2017; Valaitis et al., 2017), as well as dementia navigation programs specifically (Anthonisen et al., 2023). For example, Valaitis et al. (2017) reported an absence of partnerships with community medical and social services was a major challenge for navigators in providing their own services. Another study also identified institutional barriers, such as waitlists and a dearth of programs to navigate clients toward (Carter et al., 2017). The latter challenge has been described in the literature as “navigating to nowhere” (Anderson & Larke, 2009b, p. 27), describing navigators’ inability to match clients’ unmet needs to supports because they simply do not exist. For dementia care, particularly in rural settings, education and support services are often unavailable or inadequate (Bayly et al., 2020; Dal Bello-Haas et al., 2014; Smith et al., 2011), which matches what participants described in interviews. Furthermore, the difficulty of keeping track of a constantly changing system and siloed information, particularly around electronic medical records, are also common challenges to patient navigation programs (Carter et al., 2017; Phillips et al., 2020). In the literature on dementia navigation programs specifically, difficulties coordinating with partners and stakeholders was a common implementation barrier (Anthonisen et al., 2023). These are system level barriers that affect not only navigation programs, but also the broader population they aim to serve.

A lack of funding is a challenge for dementia care in general (Herron et al., 2016), and navigation programs for people with dementia specifically (Samus et al., 2014; Willink et al., 2020). Many dementia navigation programs that list funding as a facilitator receive that funding either from a large organization, such as the American Department of Veterans Affairs, or through grants (Anthonisen et al., 2023). This funding often necessitated strict eligibility requirements, linking program accessibility to funding. As an interviewee in this scan also reported, another downside of funding programs through grants is the uncertainty around long-term sustainability. The drawback of for-profit models is a potential lack of accessibility as people must pay for services. Notably, one participating for-profit program set some funding aside to provide services *pro bono*. One patient navigation program for people with dementia found that hiring lay navigators could contribute to saving costs (Bernstein et al., 2020). This differed from most navigators in this scan, who had professional backgrounds, such as in nursing and social work. Notably, all the patient navigation programs included in this scan paid their navigators, regardless of their funding structure.

There is a need for adequate and sustainable funding to build staff capacity and improve service quality, particularly as rates of dementia continue to grow. Increased funding would reduce many of the barriers this scan identified. It would address the precarity many organizations feel, particularly those operating on grants. A greater sense of permanence could facilitate more long-term planning, particularly around assessment and data collection to measure program effectiveness and identify gaps. It would also allow more money to hire additional staff, expand services, and extend hours of operations. Many of the organizations in this scan operate at typical business hours, during weekdays, which may not be accessible for everyone.

While this scan focused on Canadian programs, the findings have broader relevance for dementia care navigation internationally. Many countries face similar systemic barriers, including fragmented care systems, service availability and funding limitations, and geographic disparities, especially in rural or underserved regions. The barriers identified here, such as the lack of shared electronic medical records and limited funding, are not unique to Canada and impact other efforts internationally to improve care coordination and access. These insights can inform international stakeholders seeking to implement or strengthen navigation programs, highlighting the importance of investing in integrated infrastructure, tailoring services to local contexts, and ensuring equitable access across regions. Additionally, the importance of patient- and caregiver-centered approaches underscores a general need for navigation models that are responsive, adaptable, and embedded within broader health and social care systems.

## Limitations

While this study provides important foundational knowledge about dementia patient navigation programs in Canada, several limitations should be noted. Our search strategy may have missed programs. Furthermore, not all the programs identified in the scan were able to participate in interviews to contribute to qualitative analysis. We had a small sample size and as such results may not be as generalizable. Additionally, we only interviewed people delivering services, rather than clients who make use of their services, and thus our results tend to describe intended program features, which may differ from actual implementation and its effectiveness. Finally, the programs included in this study measure information, such as caseload and variations in navigator training, in different ways, complicating direct comparisons. These limitations represent ample opportunities for future research.

## Conclusion

Dementia care is not straightforward for anyone involved, be they a person with dementia, a caregiver, or a member of the care team. As the prevalence of dementia increases every year, it has been identified as a national priority in Canada. Patient navigation programs for people with dementia offer a promising approach to improving dementia care by supporting access, coordination, and continuity. This scan provides valuable insights for organizations looking to develop or implement similar navigation programs, within dementia care as well as for other populations. More widely, it can help provide guidance on potential gaps and pitfalls, as well as support for organizations, health care professionals, and decision-makers in improving integrated care and coordination.
